# Management of in- and out-of-hospital screening for hepatitis C

**DOI:** 10.3389/fpubh.2022.984810

**Published:** 2023-01-25

**Authors:** Jing Zhou, Fa-Da Wang, Lan-Qing Li, En-Qiang Chen

**Affiliations:** Center of Infectious Diseases, West China Hospital of Sichuan University, Chengdu, China

**Keywords:** hepatitis C, screening, in-hospital, out-of-hospital, special populations

## Abstract

Because of insidious progression and no significant clinical symptoms at early stage, chronic hepatitis C (CHC) is often diagnosed after the occurrence of cirrhosis and hepatocellular carcinoma. Highly effective and low drug resistance of direct-acting antiviral agents (DAAs) have enabled cure of CHC, encouraging the World Health Organization to propose a global viral hepatitis elimination program. To Date, vaccine for CHC is still under research. Therefore, reducing the source of infection is an important means of eliminating CHC other than cutting off the transmission route, which requires screening, diagnosing and treating as many patients in the population as possible. Hospital-based screening strategy have been found to be cost-effective in the management of CHC screening, as reported both nationally and internationally. Currently, China has issued *In-hospital process for viral hepatitis C screening and management in China (Draft)* in April, 2021, which provides a standardized implementation process and direction for in-hospital hepatitis C screening and treatment, but still requires medical institution to develop its own management process, taking into account its current situation and learning from domestic and international experience. In addition, screening for CHC outside the hospital among special populations, such as blood donors, pregnant women, homosexuals, intravenous drug users, prisoners, and residents in rural areas with scarce medical care resources, also requires attention and development of targeted and rational screening strategies. In this paper, we analyze and recommend the management of hepatitis C screening from both in-hospital and out-of-hospital perspectives, with the aim of contributing to the formulation of hepatitis C screening strategies.

## 1. Introduction

Hepatitis C virus is mainly transmitted through blood, and ~50–75% of infected patients develop into chronic hepatitis C (CHC) (1). CHC progresses insidiously with no apparent symptoms at early stages, and is often diagnosed when cirrhosis or hepatocellular carcinoma occurs, causing huge economic losses to both society and individuals. Therefore, CHC can be characterized as a “silent” and easily “neglected” disease.

With the availability of highly effective and low-resistant direct-acting antiviral agents (DAAs), curing hepatitis C has become a reality. In 2016, the WHO proposed in its global viral hepatitis clearance plan: Taking 2015 as the baseline, to 2030, the combined global rates of new infections and mortality of viral hepatitis B and C are to be reduced by 90 and 65% respectively. In other words, countries worldwide are required to achieve 90% diagnosis rate, 80% treatment rate and 75% reduction in the risk of occupational exposure to hepatitis C in healthcare (2).

At present, though we have developed powerful and low-resistant DAAs with SVR12 over 90%, there is no available vaccine for prevention. Therefore, elimination of hepatitis C involves two aspects: (I) reducing the reservoir of infection, including confirming patients and initiating treatment early; (II) reducing the population of new infections, including preventing infection and reinfection in uninfected and cured individuals.

Screening is the prerequisite for diagnosis, treatment, and prevention of CHC. Screening management for CHC needs to be carried out in various aspects. Prescreening preparations include investigating the epidemiological characteristics of infected population, defining the distribution of prior screening populations, identifying screening sites and testing reagents. As screening initiated, developing measures to expand screening, boost voluntary screening, and facilitate diagnosis and treatment post screening need to be considered. This review explores the characteristics and measures of in- and out-of-hospital hepatitis C screening, expecting to contribute to the formulation of hepatitis C screening strategies. At end of the review, we also summarized the deficiencies and measures in the management of hepatitis C screening in countries of different incomes (Table 1).

**Table 1:** The unmet needs and solutions to HCV screening management in countries of different incomes.

**Unmet needs**	**More fitting solutions**
**Common barriers**	
Poor awareness of HCV screening in physicians and unawareness of HCV infection status in patients contribute to continued HCV transmission and missed prevention.	Adopt risk-based HCV testing in low- or middle-income countries, universal testing to targeted groups or general population in high-income countries.
	Promote periodly awareness campaigns for the public, educate at-risk populations and patients, provide professional training to health care workers.
Substantial participants do not link to care after screening.	Streamline the procedure of HCV cascade cure.
	Well-trained general practitioners charge for patients' screening, diagnosis and therapy.
**Unique Barriers**	
**Low-and middle-income countries**	
Limited health-care resource and insurance coverage schemes induce poor access to HCV test.	Request assistance from non-government foundations.
A high proportion of individuals with HCV infection remain undiagnosed.	Adopt micro-elimination strategy in targeted populations, with gradual transition to universal screening.
The lack of laboratory equipment and technology restricts the advantages of NAT. HCV screening of blood donors is inadequate.	Choose easy-to-operate and highly sensitive screening assays, such as ELISA for serum antibody.
Universal screening is rarely actualized before and during pregnancy.	Provide professional training to general practitioners.
	Work with obstetricians and select highly sensitive HCV antibody tests to promote testing for women of childbearing age or pregnancy.
**High-income countries**	
Injection drug use (IDU) and Men who have sex with men (MSM) remain the key risk factor for new hepatitis C infections.	Promote HCV testing in PWID and increase access to harm reduction programs, such as needle exchange programs.
	Implement national HCV register to track progress and yearly screening.
Chances for screening are plentiful but no adequate linkage to care for sufficient treatment.	Take a holistic approach to HCV elimination with coordinated screening, linkage-to-care and treatment efforts.

## 2. In-hospital hepatitis C screening

### 2.1. Necessity of in-hospital hepatitis C screening

Anti-HCV screening within hospitals is low cost and effective, helping to find people with HCV infection. Studies have shown that the hepatitis C antibody screening rates in tertiary hospitals is high, and the antibody positivity rate is also higher than that of the general population. Data collected by Niu et al. indicated that the hepatitis C screening rate of inpatients in China's top three hospitals exceeds 50%, and the average positive rate of hepatitis C antibodies is 0.88%, which is higher than the prevalence rate of the general population (0.43%) (2). In a study, conducted during 2015-2016, four urban emergency departments (ED) in the USA adopted opt-out, universal hepatitis C screening for 14,252 patients and 1,315 (9.2%) had positive test results for anti-HCV. Prevalence of positive results for HCV RNA at the four ED sites was 5.7%, which was substantially higher than the overall U.S. prevalence of positive results for HCV RNA of 0.95% (3). A UK study demonstrated that the higher the prevalence of hepatitis C, the more cost-effective screening is (4).

However, in primary care settings, many patients are missed without adequate HCV screening (5). At the same time, many healthcare workers lack awareness of further management of antibody positive individuals, particularly in some developing countries, primary care facilities and non-specialist departments. Data from a tertiary hospital in Turkey revealed, in anti-HCV positive patients, HCV RNA testing was requested least by surgery and other clinics (21 and 25% respectively), while most by infectious disease (100%) and gastroenterology (70.58%) clinics (6). In addition, although hepatitis C screening rates are high in hospitals, further diagnosis, referral and treatment rates are low, which is not uncommon in developed countries. In order to eliminate hepatitis C, George ley Asia in the United States launched a nationwide project to screen hospitalized patients for free hepatitis C antibodies in November 2016 (7). Through the analysis of data after 1 year, it found, overall, the screening rate was as high as 86.6%, 4.9% of patients screened positive, but only 19.8% of eligible anti-HCV positive patients were linked to care. Thus, low linkage-to-care rates underscore the need for screening programs to be coupled with effective linkage strategies.

Inpatients tend to be more health-conscious. Therefore, if hospitals can provide a one-stop service for screening, diagnosis and treatment, the ineffective and repeated screening can be avoided to a certain extent. Early diagnosis and treatment may increase medical expenses in the short term, but can reduce the long-term medical costs of hepatitis C-related complications. Therefore, standardizing the process of in-hospital hepatitis C screening is not only taking advantage of the unique advantages of hospitals, but also improving its shortcomings and the “deficiencies” of untimely post-screening treatment.

### 2.2. Recommendations for in-hospital hepatitis C screening

China is one of the five developing countries with the largest burden of hepatitis C in the world. In order to practice the hepatitis C clearance target proposed by the WHO in 2016, medical institutions at all levels should attach importance to correctly and reasonably implementing of hepatitis C testing (8, 9). However, specific screening program needs to be adjusted according to the actual situation of each medical institution, and constantly explored and innovated in practice. Here, we provide summary recommendations that should be emphasized from the following three aspects.

#### 2.2.1. Co-education for three groups

The “three groups” refers to medical staff, patients and uninfected groups. Based on increasing testing awareness, “co-education” emphasizes adopting multiple models, personalized content, and continuity.

Specialized training should be conducted for physicians, especially those in primary hospitals and non-hepatology specialties, to increase their knowledge on the disease, to inform them about the insurance reimbursement policy, to enhance their awareness of screening, as well as their sense of responsibility and mission. For example, organizing knowledge delivery from hepatologists in non-specialized departments, carrying out knowledge lectures from experts at primary hospitals. However, in the era of rapid development of Information and Communication Technology (ICT), a modern way advancing with the times is to establish web-based resource centers for primary care providers. Through such online platforms, general practitioners have easier access to real-time updated knowledge, and easier access to the guidance from experts.

For the uninfected population, education is intended to raise awareness of prevention, by covering the transmission routes of HCV and specific preventive measures, and encourage people with high risk to engage in proactive and regular screening. Another aim is to correct social prejudice and discrimination against patients. For those with positive antibodies, education should focus on highlighting the terrible complications of hepatitis C and urging early diagnosis. For diagnosed patients, we should not only inform them the harms of hepatitis C, but also highlight the curability of diseases and the reimbursement of expenses. For cured patients, we should focus on highlighting the risk of reinfection and the necessity of regular examination to monitor liver conditions.

Finally, hepatitis C education is not a one-off, but a long-term work. We must utilize various information platforms, grasp the characteristics of the target audience, innovate the delivery mode of education, and obtain the participation of people through multiple methods before the harm and prevention of hepatitis C can be deeply rooted in people's hearts.

#### 2.2.2. Grasping the principles and completing cascade cure

The hospital-based hepatitis C screening process has institutional variability, but should share common principles: patient-centeredness, streamlined process, proactive screening, and outreach screening.

Patient centricity means that in the absence of a mandatory screening policy, sufficient communication should be made with the patient to obtain consent for screening, to understand their concerns and difficulties, to provide psychological support and treatment guidance, to help obtain social assistance, and to reduce barriers to cascade cure.

Simplifying the process is designed to facilitate completion of the hepatitis C cascade cure. Feasible measures include: shortening the test reporting time and advising antibody-positive patients and physicians to complete HCV RNA testing by expeditious means, such as phone call or performing reflex RNA assays directly by the laboratory department. The optimal solution is to create green passage to provide one-stop service for screening, diagnosis and treatment. The measure contribute to eliminate the situation of delaying treatment for patients' intimidation and bewilderment of tedious registration and cumbersome process, to improve patient compliance and to reduce case loss rate. Lower-level medical institutions unable to confirm diagnosis, are obliged to refer patients with positive antibody to the nearest, reliable higher-level medical institution.

As screening awareness is lacking among many physicians and patients, in actual clinical practice, preparations for proactive screening can be up-front. For example, in advance of the physician visit, distributing questionnaires for screening high-risk groups and playing educational videos. During the visit, alert signals will be sent through the electronic medical record system once the targeted population appears, reminding physicians to advise patients completing screening and make an appointment for the next visit. For high-risk patients who refuse screening, follow-up can be done by specialized medical staff, such as a nurse, to provide ongoing education and repeated recommendation of screening.

When medical resources permit, hospitals should be encouraged to implement universal testing. Considering to identify more asymptomatic infections and high-risk populations with concealed medical history, screening should be carried out to those who desire and ought to be screened, rather than only focusing on high-risk populations.

#### 2.2.3. Intellectualizing the hospital information system and establishing screening management network platform

The design of intelligent electronic medical record system should start from the logic of reducing the clinical workload and improving work efficiency. For instance, if the record system can automatically display the current hepatitis C screening, diagnosis and treatment status of patients, and can send correct and standardized diagnosis and treatment instructions to physicians, this intelligent change will help to reduce the probability of over-screening, missed examination or delayed treatment.

Establishing a real-time database for HCV screening should be taken into account. It will be convenient for physicians to query and upload the infection information of the patients, to make real-time choices for further diagnosis and treatment, and to avoid repeated screening and ineffective screening. It will be conducive for the Center for Disease Control and Prevention to master local hepatitis C clearance in real time, and conducive for the relevant departments to adjust the HCV elimination policies.

Take the HCV Sicily Network for example. It is a web-based model designed to improve the management and treatment of HCV chronic hepatitis and cirrhosis. Total 41 clinical centers and 101 specialist physicians (gastroenterologists, hepatologists, infectivologists, internal medicine physicians) are involved in the platform. The general practitioners (GPs) and hospital specialists are key players in the correct management of patients with chronic HCV infection. Through the web site, GPs can make direct communication with hospital specialists, book online expert outpatient visits directly, and track patients' diagnosis-treatment path. Moreover, this network continually provides with highly precised data on the efficacy and tolerability of antiviral therapies to the regional health organization and the scientific community. According to the statistics, From March 2015 to December 2018, 16,500 patients have been recorded in the web platform, 12,300 completed the treatment. Among the patients treated, >90% achieved SVR12. The rate of SVR12 was 95.1% in patients with chronic hepatitis, 93.2% in Child–Pugh A cirrhosis (93.2%), and 82.2% among those with Child–Pugh B cirrhosis. In the future, such an excellent telemedicine platform will be universally popular around the world, and more important elements may be added to it, such as psychologists, charitable organizations, to make such platforms more humane and professional (10, 11).

## 3. Out-of-hospital screening

As already mentioned, hospital-based hepatitis C screening is a centralized screening mode with the advantage of high screening rates and low cost, and is an important way to identify potentially infected individuals. Because hepatitis C can be asymptomatic for long periods, and for other reasons such as economic hardship, transportation isolation, disease stigma, and low health awareness, there are some people who do not appear in the hospital or do not have access to the full range of physician guidance. Therefore, it is necessary to focus on populations that are difficult to reach through hospital screening. Here, the main out-of-hospital screening populations discussed include blood donors, marginalized populations of society (including intravenous drug users, prisoners and male-male homosexuals), pregnant women and rural residents in remote areas.

### 3.1. Blood donors

Blood transfusion is one of the critical ways of HCV transmission, and screening blood donors can reduce the risk of transfusion-transmitted HCV. The safety hazards of blood transfusion are mainly originate from window periods, rare subtypes, viral variants and immune silence leading to false negative test results. However, the limited advancement and sensitivity of testing technology and the existence of a testing window make post-transfusion residual risk (RR) unavoidable. Residual risk (RR) refers to the possibility that after a blood donor is screened for blood-borne viruses, the recipient may be infected with blood components that are “qualified” for blood safety screening.

Currently, screening pattern prevailing in developed countries is enzyme-linked immunosorbent assay (ELISA) or chemiluminescence immunoassay (CLIA) for detecting HCV antibodies, combining nucleic acid amplification technology (NAT) for HCV RNA screening, where the residual risk of blood transfusion is controlled at a very low level. Throughout the 1990s, the risk of transfusion-transmitted (TT) hepatitis C was declined rapidly to 0.01% (per unit transfused) by introduction of ELISA to detect anti-HCV at blood centers (12). Over the last decade, the adoption of HCV NAT at blood centers, along with the use of improved screening reagents and strict donor selection procedures, the risk of TT-HCV in the United States was further declined to 0.0001% (per unit transfused) (13). Both serological testing and molecular nucleic acid testing have their advantages and disadvantages. It is wise to combine the two and compensate each other in order to maximize the safety of blood products. As for serological tests, the advantages are high sensitivity, low cost, and easy operation, however, the drawbacks are the long detection window, which is as long as 66 days even with the improved third generation reagent, and the possibility of false positives/false negatives. NAT can significantly shorten the detection window, but may still yield false negative outcomes and lead to missed tests in cases with low viral load or viral genetic mutations. Furthermore, this assay is limited in some developing countries due to its high cost and the needs for laboratory equipment, instruments, and personnel (14).

What actions can be taken to meet the challenges for developing countries? Currently, there are still countries in the world that do not routinely screen for HCV, and in some African countries with high HCV prevalence, antibody screening is performed using the less sensitive rapid detection tests (RDTs), which means that a significant proportion of HCV-infected blood products are unsafe. Countries with scarce resources need to develop screening programs that are low-cost and relatively safe in light of national circumstances. First of all, blood donors should be recruited from low-risk groups and the test operation process should be standardized. Countries without routine pre-donation HCV screening should take actions as early as possible. even with less sensitive rapid detection tests can reduce the transmission of HCV to some extent (15, 16). When HCV RNA testing is not available, the use of HCV antibody tests from two different manufacturers or the use of cheaper antibody combined with antigens may be the first option, instead of rapid screening test (14, 17). In a study by Syria et al. (14) blood donors were tested simultaneously for HCV markers by routine antibody test based on rapid test and HCV antigen/antibody combination assays. They found the later showed a better sensitivity of 91.9%, higher than that of antibody test (70.3%), supporting the implementation of Ag/Ab combined test in the African blood bank setting (14). Laperche et al. (18) reported Monolisa HCV Ag/Ab assay reduced the window period by 26.8 days (range, 0–72 days) which is on average 5.1 days (range, 0–24 days) later than NAT (18). In Schnuriger's study, they covered the combined assay became positive as early as the first PCR and earlier than a third-generation enzyme-linked immunosorbent assay in 65% of the HIV infected patients who suffered from acute hepatitis C, improving the diagnosis of hepatitis C infection, especially in high-risk populations (19). If the screening setting does not allow for antibody/antigen testing, pre-donation screening can be performed with RDTs, then the blood with negative antibody can be collected with the aid of dried blood spot (DBS) and transported to laboratories for a more accurate screening test (20). Ideally, of course, widespread use of ELISA combined with NAT screening at blood stations would be desirable whenever possible.

Finally, for individuals with positive HCV antibodies or HCV RNA, on the one hand, it is necessary to provide psychological counseling, reduce stigma and popularize the knowledge of hazards of hepatitis C, to promote the next step of consultation. On the other hand, information on those should be uploaded into the HCV screening management database for follow-up and reducing the rate of repeat screening.

### 3.2. Pregnant women

Pregnancy with hepatitis C infection is associated with an increased risk of mother-to-child transmission, increased rates of preterm birth and late mortality in newborns, and a 20-fold greater risk of intrahepatic cholestasis than those uninfected (21). The exact mechanism between HCV infection and intrahepatic cholestasis of pregnancy (ICP) is unclear. Some hypothesized explanations are the direct cytopathic effect from persistent HCV viremia, variant alleles of ABCB11 (ATP-binding cassette, subfamily B, member 11) gene, and dysfunction of the principal sinusoidal or canalicular bile acid transporters caused by HCV infection and high estrogen and progesterone levels during pregnancy (22–25). In addition, unlike hepatitis B, there are no measures to block mother-to-child transmission of hepatitis C, and no drug now has been approved for DAAs in pregnancy.

Preconception is the best time for screening, so universal, mandatory screening for women of childbearing age is necessary. If missed, universal screening should also be implemented during pregnancy, and at every pregnancy (26). It has been shown that universal screening improves the detection of HCV infection in pregnancy than risk factor-based screening, and is more cost effective (27–29). Identifying the HCV infection status of pregnant women helps obstetricians to manage their patients. Because of the frequent contact between pregnant women and physicians during pregnancy, which will facilitate physician referral and hepatitis C awareness and education, thus increasing the rate of treatment after delivery (30). Actually, some pregnant women have the desire to initiate treatment during pregnancy.

Similar with the general, HCV screening tests during pregnancy are routinely performed with antibody and HCV RNA screening. However, due to the cost and accessibility of test, universal screening for HCV during pregnancy is only available in a few countries and failed in low- and middle-income countries (26). Therefore, economic and policies are needed to support the implementation of HCV screening before and during pregnancy.

### 3.3. Poor rural areas

Compared with urban areas, screening rate of HCV is lower in rural areas (31–33). Scarcity of medical resources, transportation congestion, and proximity to medical service centers, all reduce the availability and accessibility of basic medical services for rural residents. Economic income constrains the affordability of medical services for rural residents. The cultural background of the community may influence the recognition and beliefs about the necessity of hepatitis C screening. Poor health awareness and insidious progression of chronic hepatitis C delays clinic visits (32).

Therefore, increasing hepatitis C testing in countryside, it depends on expanding education to change people's cognition and awareness, to change people's misunderstanding and prejudice against hepatitis C, and to change patients' self-stigma. It depends on financial compensation and free screening to motivate residents' engagement, and create environment for active screening. It depends on expanding medical reimbursement and increasing the ratio of medical resources per capita. It depends on transiting to decentralized screening by bringing tests to family physicians and rural clinics, or using telehealth, mobile health care units to universalize Screening (34). Gamal Shiha et al. implemented successfully a comprehensive community outreach program, on a village with high burden of HCV infection in north Egypt, which consisted of community mobilization, educational campaign, fundraising for public donations and comprehensive testing. At the end, the proportion of participants with a good general awareness and understanding of HCV transmission increased from 27% to about 70%, and 89% (4215/4721) of eligible villagers were screened for HCV antibodies (35).

### 3.4. PWID, MSM, and prisoners

The three groups, people who inject drugs (PWID), men who have sex with men (MSM) and prisoners, are high-risk populations of HCV infection and the main sources of infection. The exposure to HCV reinfection and co-infection with HIV is high due to high-risk behaviors, such as intravenous drug use, condomless sex, and tattoos. In some cases, antibody screening is not effective, such as early diagnosis of acute reinfection, or co-infection with HIV resulting in delayed or no HCV antibody generation. At this circumstance, direct testing of HCV core antigen, which costs less than HCV RNA test, might accelerate early diagnosis and prevent onward transmission (36, 37).

Prisoners have low security of ongoing medical care because of restricted personal liberty, but the relatively closed environment in prison makes it easier to conduct screening. In contrast, the difficulty of access is the main obstacle to screening the drug-addicted population (38). For the former, change in political will is needed, to ensure accessibility of hepatitis C screening in prisons. For the latter, hospitals offering opioid substitution therapy (OST), prisons and needle/syringe exchange sites, and detoxification clinics can serve as breakthroughs for screening. Additionally, measures can be taken involve creating community-based screening programs, establishing counseling websites, and encouraging PWID to bring their friends and injection partners to screening (39). For MSM, it has been shown that HCV screening information could be delivered by private message on gay applications (APPs), which has been shown to be acceptable and effective (40). HCV infection is more common in MSM with co-infection with HIV, therefore, HCV screening reagents, such as On-site rapid HCV antibody testing, can be given in prevention and treatment service sites for HIV.

In general, screening of these three groups should be based on providing financial supports, fighting self-shame, raising awareness of regular screening, increasing their trust in national policies and social assistance, giving them tolerance and patience, and avoiding isolation, discrimination and marginalization, so as to effectively reduce the risk of re-infection and reduce the source of infection.

## 4. Conclusion

Hepatitis C screening and management is a sustained and collaborative battle that requires brainstorming, optimizing techniques and taking actions. Future initiatives to accelerate HCV elimination are, expanding access to community-based testing using HCV point-of-care tests among at-risk and general populations; adopting decentralized and integrated HCV one-stop services at harm reduction sites, detention settings and primary care; expanding treatment to include children and adolescents; addressing stigma and discrimination; and ensuring sustainable financing through domestic resources to scale-up testing, treatment and prevention.

To eliminate the harm of hepatitis C, screening is the foundation and prerequisite to ensure the completion of the goal, and is the first step among many obstacles. Solutions to the barriers of screening, require government leadership, a commitment to hepatitis C eradication, financial input, policy reform and practice; require the wisdom and strength of all sectors of society, and multi-pronged plans; require conducting national and regional epidemiological surveys of hepatitis C, investigating the challenges to screening of different populations, encouraging to innovate screening models, and formulating practical and feasible hepatitis C screening plans in line with national conditions. Through scientific plan and practical implementation of relevant measures and strategies, we have reason to look forward to the day when hepatitis C becomes globally eliminated.

## Author contributions

JZ and E-QC: proposed the ideas, designed, and wrote manuscript. F-DW and L-QL: searched literatures and assisted in conducting the manuscript revision. All authors contributed to the article and approved the submitted version.
